# Bathymetry of the Philippine sea with convolution neural network from multisource marine geodetic data

**DOI:** 10.1016/j.isci.2025.114285

**Published:** 2025-11-28

**Authors:** Jia Guo, Shuai Zhou, Jinyun Guo

**Affiliations:** 1 INRIA, 06902 Nice, France; 2 State Key Laboratory of Precision Geodesy, Innovation Academy for Precision Measurement Science and Technology, Chinese Academy of Sciences, Wuhan 430077, China; 3 College of Earth and Planetary Sciences, University of Chinese Academy of Sciences, Beijing 100049, China; 4 College of Geodesy and Geomatics, Shandong University of Science and Technology, Qingdao 266590, China

**Keywords:** earth sciences, oceanography, geodesy, machine learning

## Abstract

This study developed a deep learning-based method for high resolution bathymetry prediction in the Philippine sea, aiming to improve the accuracy of seafloor depth estimation using multi-source marine geodetic data. The method integrates geographic coordinates with auxiliary features, such as bathymetry, sea-land marks, seafloor slope and orientation, gravity anomaly, vertical gravity gradient, mean dynamic topography, deflection of the vertical, mean sea surface, and sedimentary thickness. These inputs were extracted from an 8 × 8 arcminute region around each training point and the model was trained to predict depth residuals. The trained model was applied to 1 × 1 arcminute grids to generate a detailed bathymetric map. Evaluation against ship-borne measurements shows that the developed method significantly improves prediction accuracy compared with existing models of similar resolution and performs comparably to a state-of-the art high-resolution model. This work demonstrates the potential of deep learning to enhance large-scale, cost-effective seafloor mapping using widely available geophysical datasets.

## Highlights

• Deep learning model for high resolution bathymetry in the Philippine sea
• Integrated multi source geodetic data to enhance accuracy
• Surpassed similar models and rivaled higher-resolution models

## Graphical abstract

**Figure:**
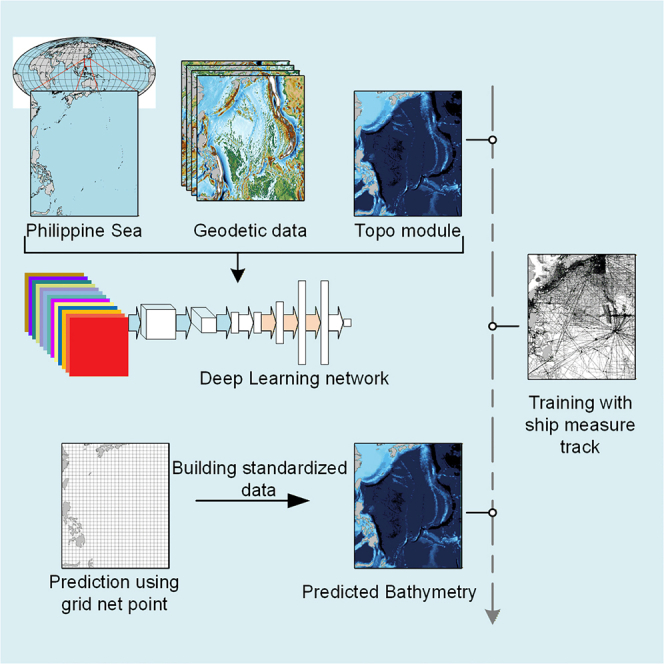

## Introduction

**Figure 1:**
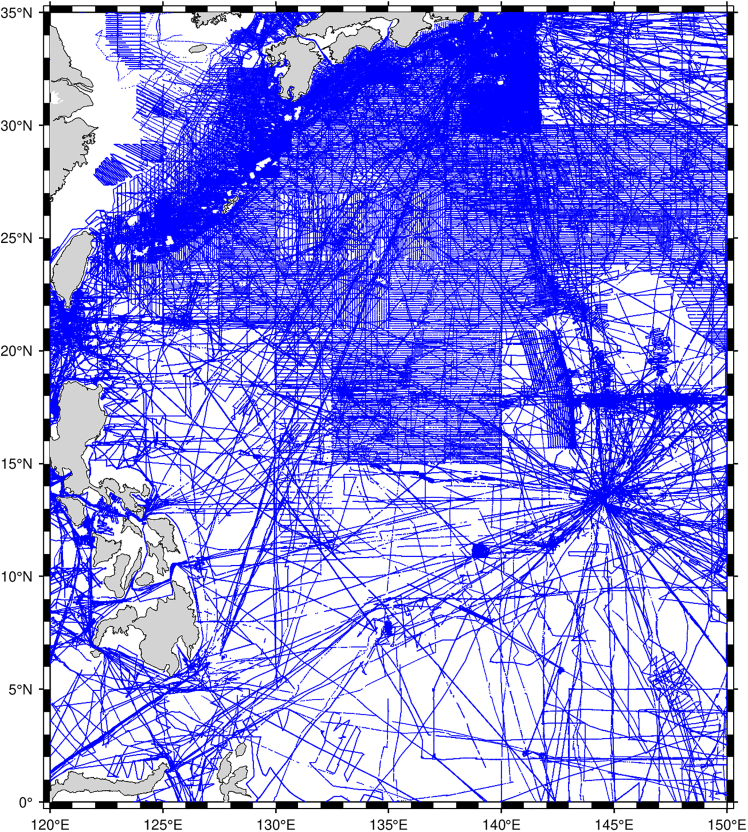
Geographical location of the selected study area in the Philippine sea and the distribution of ship-borne single-beam bathymetry data. The blue dots are the bathymetric measurements points

The Philippine sea, located in the western Pacific ocean near the Philippine islands (Figure 1), forms part of the tectonically active Pacific rim seismic belt. The region exhibits a highly complex structure resulting from the dynamic interactions among the Philippine sea, Eurasian, and Pacific plates.^1^^,^^2^^,^^3^ These plate convergences^4^^,^^5^^,^^6^ have led to diverse seafloor topographies, including deep oceanic trenches (e.g., the Mariana Trench), extensive seamount chains, sedimentary basins and intricate ridges,^7^^,^^8^ typical of a trench-arc-basin system.

Accurate and high-resolution bathymetric mapping is crucial for a wide range of marine scientific research,^9^^,^^10^^,^^11^^,^^12^^,^^13^^,^^14^^,^^15^^,^^16^ including ocean current analysis,^9^^,^^11^^,^^13^ seafloor topography modeling,^10^^,^^12^ environmental monitoring,^14^ and marine conservation.^16^ Given these scientific, ecological, and economic implications, accurate mapping of seafloor depth known as bathymetry, is of fundamental importance. Traditional methods of bathymetric data collection include ship-borne sonar systems, airborne LiDAR bathymetry and satellite altimetry-based gravity inversion. Ship-borne techniques measure ocean depth directly with high precision using single-beam and multi-beam echo-sounder systems, however, they are time-consuming and costly.^17^^,^^18^ Given the vast extent of the ocean, obtaining complete bathymetric coverage remains challenging.^19^ Bathymetric measurements are highly dependent on ship’s route, resulting in sparse and uneven spatial coverage. Airborne LiDAR offers high measurement efficiency; however, its application is limited to shallow waters (typically less than tens of meters deep) and its performance is highly sensitive to environmental influences like weather conditions.^20^^,^^21^^,^^22^ With ongoing advances in satellite altimetry, marine gravity data derived from sea surface height measurements have reached high resolution and accuracy.^23^^,^^24^^,^^25^ These gravity data have been widely used to derive seafloor topography models.^26^^,^^27^ Remote sensing bathymetry estimates water depth by analyzing the spectral reflectance of shallow waters, particularly using blue and green bands.^28^

Bathymetry prediction has traditionally relied primarily on satellite altimetry-derived gravity data, with various inversion techniques being developed over the years. Representative methods include the Smith-Sandwell method,^29^ the simulated annealing method,^30^ the admittance function method,^31^ the least squares method^32^ and the gravity-geologic method.^26^^,^^33^ However, these methods face significant limitations, as they typically rely on single type data. Traditional models often require region-specific calibration and substantial manual intervention, which limits their scalability and potential for automation.

Recent advances in marine technology and artificial intelligence (AI) have created new possibilities for enhancing bathymetric mapping. Machine learning and deep learning algorithms, in particular, have demonstrated remarkable capabilities in processing large datasets, identifying patterns, and making accurate predictions.^34^^,^^35^^,^^36^^,^^37^ These technologies offer significant potential to improve both the efficiency and accuracy of bathymetric mapping by leveraging existing data and capturing complex patterns often missed by traditional methods.

This study employed a convolutional neural network (CNN) applied to ship-borne bathymetry data and multi-source marine geodetic datasets including gravity anomaly, vertical gravity gradient (VGG), mean dynamic topography (MDT), deflection of the vertical (DOV), mean sea surface (MSS), and sedimentary thickness to establish a bathymetry model of the Philippine sea region (0°-35°N, 120°-150°E) at a 1 arcminute (arcmin) resolution. The performance of the developed method was evaluated through comparisons with existing bathymetric models, including the Topo_25.1,^38^ ETOPO_2022,^39^ and GEBCO_2024^40^ models.

## Results and discussion

In order to assess the accuracy of the model, we employed Topo_25.1, ETOPO_2022, and GEBCO_2024 model, which were interpolated to the ship-borne bathymetry test points. As shown in Table 1, the model achieved the lowest mean error (0.41 m) which is comparable to that of ETOPO_2022 (−1.29 m) and the lowest standard deviation (STD) (44.90 m), indicating a centered and stable prediction compared to Topo_25.1, ETOPO_2022, and GEBCO_2024. Notably, the error range of the developed method was substantially narrower, with maximum and minimum errors constrained within 1100 m, whereas GEBCO_2024’s error spanned over 3700 m.

**Table 1:** Performance metrics of three bathymetric models at test points

	Max (m)	Min (m)	Mean (m)	STD (m)	RMS (m)	R^2^ (%)	MAE (m)
Topo_25.1	209.14	−188.10	7.59	54.34	54.87	99.932	35.03
ETOPO_2022	1930.79	−1656.32	−1.29	54.03	54.05	99.934	27.31
GEBCO_2024	1930.56	−1817.31	1.42	54.43	54.45	99.933	26.81
Developed method	449.10	−699.32	0.41	44.90	44.90	99.955	28.51

MAE, mean absolute error; RMS, root-mean-square error; R^2^, coefficient of determination.

Further evaluation using multiple statistical metrics is presented in Table 1. The method achieved the best performance in terms of root-mean-square (RMS) (44.90 m), coefficient of determination (R^2^) (99.955%), while yielding a mean absolute error (MAE) of 28.51 m. While the MAE is slightly higher than those of ETOPO_2022 (MAE = 27.31 m) and GEBCO_2024 (MAE = 26.81 m), it remains lower than that of Topo_25.1 (MAE = 35.03 m). In terms of robustness, 98.09% and 99.98% of the predicted points from the method fall within absolute error thresholds of 150 m and 300 m, respectively, reflecting both accuracy and reliability across a wide spatial range.

These results collectively suggest that the developed method achieves a better balance between accuracy and stability, making it a suitable choice for large-scale bathymetry prediction tasks.

### Over accuracy assessment

**Figure 2:**
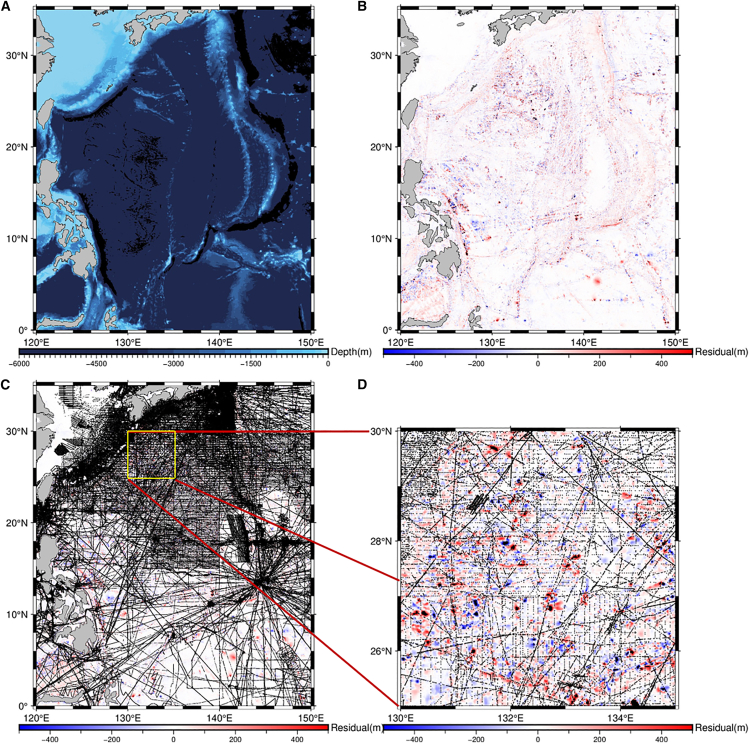
Bathymetric models and error analysis of the selected region (A) Bathymetric model generated by the developed method, where the bar indicates water depth (m); (B) Differences between the developed method and Topo_25.1 model, where the bar indicates bathymetric differences; (C) Differences between the developed method and Topo_25.1 model overlaid with ship-borne training data points (black dots), using the same scale as in (B); (D) Magnified view of a representative sub-region (130°E−135°E, 25°N–30°N), using the same scale as in (B–C).

Figure 2 presents the constructed bathymetry model generated by the developed method and its differences relative to the Topo_25.1 model. The spatial distribution of residuals (Figure 2B) reveals that most differences fall within ±400 m, with blue and red regions indicating shallower and deeper predictions, respectively. Areas beyond the display boundary are masked in black. In the overall view of the difference map (Figure 2C), linear striping patterns are visible throughout the domain. A closer inspection of the region 25°–30° N, 130°–135°E (Figure 2D) demonstrates that these patterns spatially coincide with the shipborne training data tracks.

This spatial correlation suggests that the residual artifacts may be partially influenced by the spatial bias in training data, which is concentrated along discrete survey lines. While the developed method mitigates global errors, some localized overfitting or memory effects may persist along heavily sampled paths, resulting in structured residual bands.

### Performance under different depth intervals

To investigate the developed model’s performance across different water depth zones, we also analyzed prediction errors within five water depth intervals: 0–1000 m, 1000–2000 m, 2000–3000 m, 3000–4000 m, and >4000 m. Across all depth intervals, the developed method consistently delivers accurate predictions than Topo_25.1, ETOPO_2022, and GEBCO_2024 (Table 2). The developed method consistently achieves lower RMS and higher R^2^ across all depth intervals, with mean differences close to zero, demonstrating superior stability and accuracy. It clearly outperforms Topo25.1 and ETOPO_2022 at all depths and shows comparable or even better performance than GEBCO_2024, particularly in deep and ultra-deep waters. This comparison indicates that the results generated by the developed method align more closely with ship-borne bathymetric measurements than those of the other models.

**Table 2:** Performance comparison of Topo_25.1, GEBCO_2024 and the developed method across different water depth intervals at test points

Depths (m)	Number of test point	Model	MEAN (m)	STD (m)	RMS (m)	R^2^(%)
0∼1000	324790	Topo_25.1	9.50	56.95	57.73	96.426
		ETOPO_2022	−5.70	41.08	41.47	98.156
		GEBCO_2024	−0.35	40.98	40.98	98.199
		Developed method	1.16	38.33	38.35	98.423
1000∼2000	135155	Topo_25.1	2.47	52.04	52.10	96.642
		ETOPO_2022	1.03	60.18	60.19	95.519
		GEBCO_2024	3.28	59.57	59.66	95.597
		Developed method	0.04	43.69	43.69	97.639
2000∼3000	112041	Topo_25.1	7.89	52.83	53.42	96.698
		ETOPO_2022	5.03	65.54	65.73	94.999
		GEBCO_2024	4.39	64.82	64.96	95.116
		Developed method	2.42	52.75	52.81	96.554
3000∼4000	107675	Topo_25.1	11.45	53.76	54.97	96.225
		ETOPO_2022	7.51	70.11	70.51	93.787
		GEBCO_2024	6.74	71.97	72.28	93.471
		Developed method	0.94	52.51	52.51	96.554
>4000	237233	Topo_25.1	5.55	51.22	51.52	99.675
		ETOPO_2022	−0.14	58.59	58.59	99.579
		GEBCO_2024	0.55	60.55	60.55	99.550
		Developed method	−1.67	49.99	50.01	99.693

RMS, root-mean-square error; R^2^, coefficient of determination.

**Figure 3:**
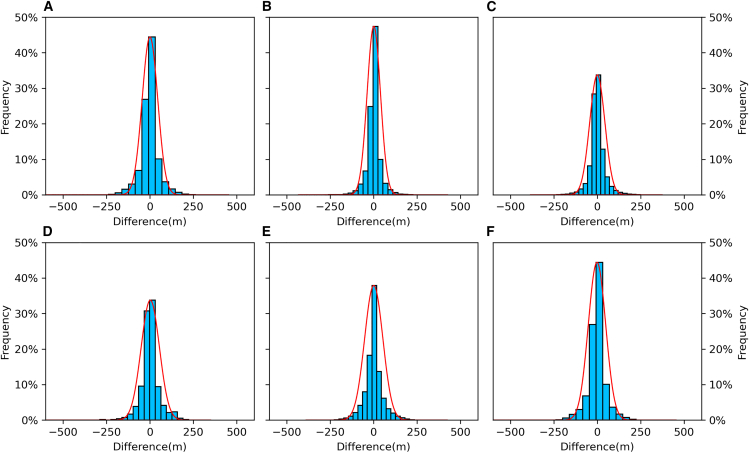
Histograms of difference distribution between ship-borne bathymetry and predicted depths across different depth intervals (A) all depth, (B) [0–1000 m], (C) [1000–2000 m], (D) [2000–3000 m], (E) [3000–4000 m], (F) [>4000 m]. The red lines represent the fitted normal distribution curves.

The histograms (Figure 3) illustrate the distribution of prediction errors in each depth interval defined in Table 2, with each subfigure corresponding to a respective depth range. Red lines represent fitted normal distribution curves. The developed method shows error distributions that are centered closely around zero for all depth intervals, with symmetric and narrow Gaussian-like shapes. In shallow and intermediate regions, the histograms exhibit relatively higher peak frequencies and lower spread, suggesting reduced variance and tighter prediction control. The error distribution in 1000∼3000 m region (Figures 3C and 3D) remains highly concentrated within ±100 m, highlighting the model’s effectiveness in areas that are often challenging for conventional datasets.

**Figure 4:**
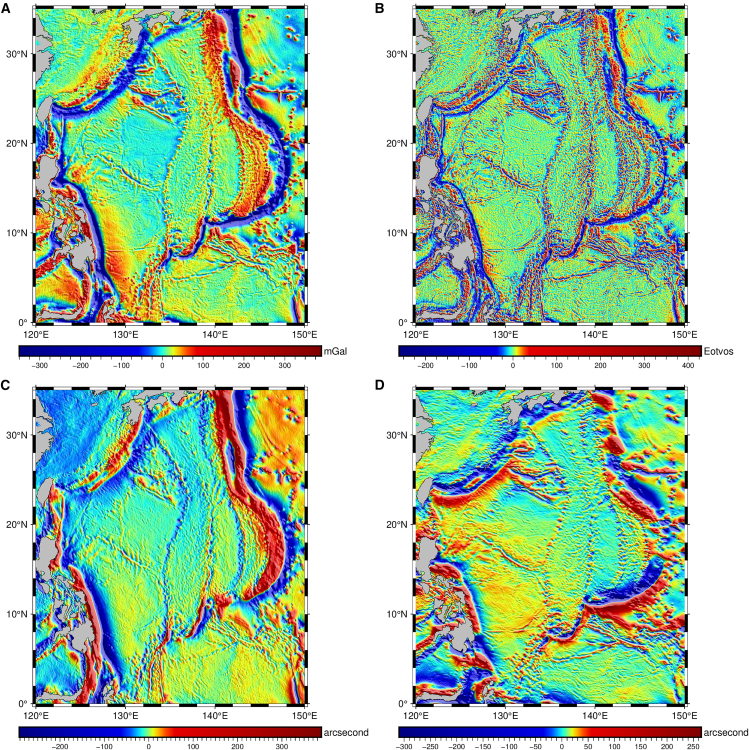
Spatial distribution of auxiliary geophysical variables used for bathymetry prediction in the Philippine sea (A) Gravity anomaly model SDUST2022GRA. (B) Vertical gravity gradient. (C) Meridian component of deflection of the vertical. (D) Prime component of deflection of the vertical.

**Figure 5:**
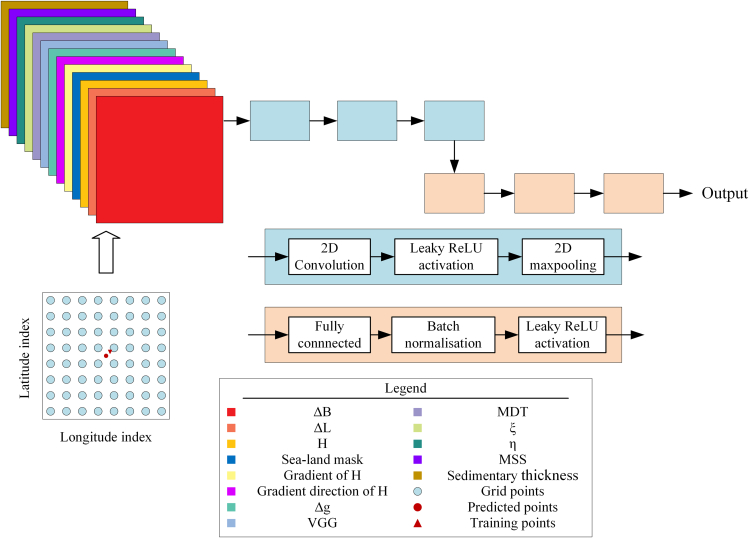
Architecture of the developed CNN and structure of the input feature grid ΔB and ΔL present differences of longitude and latitude, H presents the bathymetry from Topo_25.1 model, Δg present the gravity anomaly, ξ and η present the meridian and prime components of DOV.

In deep water regions (>3000 m), the spread increases slightly, which is expected due to reduced data density and terrain complexity. However, even in these deeper regions, the model maintains a well-centered and nearly symmetric error profile, and outliers remain rare, confirming the robustness and stability of the prediction process.

The results demonstrate that the developed method outperforms all the three models, Topo_25.1, ETOPO_2022, and GEBCO_2024 across all depth intervals, particularly in deeper regions where traditional gridded datasets often suffer from sparse sampling and interpolation artifacts. The improved accuracy and reduced error variance across the entire depth range highlight the model’s ability to generalize spatial features beyond the training distribution. The symmetric and Gaussian-like residual distributions indicate that the model avoids systematic bias and captures the seafloor morphology with minimal distortion. These findings are significant, as they suggest that the developed method can serve as a reliable tool for high-resolution bathymetric reconstruction.

In conclusion, we developed and evaluated a CNN model for predicting bathymetric depth using multi-source marine geodetic data at a 1 arcmin resolution, focusing on the Philippine sea. To improve prediction accuracy across various depth ranges, input features included sea-land masks, bathymetry variation trends represented by gradients and directions, sedimentary thickness, and additional geophysical variables.

By comparing the predicted bathymetry from the developed method, Topo_25.1, ETOPO_2022, and GEBCO_2024 models with ship-borne bathymetry data at test points, a notable improvement in the precision was achieved using the developed method (with mean of 0.41 m and STD of 44.90 m).

Furthermore, the accuracy of these models was evaluated across different water depth conditions. The results show that the developed method outperforms the Topo_25.1 and ETOPO_2022 models across all depth regions, and obtain a similar performance comparing with GEBCO_2024 model. These findings demonstrate the reliability of the developed method across various water depth ranges.

All these verifications demonstrate that the developed model, incorporating multi-source marine geodetic data, improves accuracy beyond that of models with similar resolution, achieving performance comparable to higher-resolution models. These results highlight the potential of deep learning-based approaches for large-scale, cost-effective bathymetric mapping. This work provides a scalable framework that can be extended to other oceanic regions and contributes to advancing data-driven marine geodesy.

### Limitations of the study

Although the CNN-based approach demonstrates promising performance in bathymetry prediction, its generalization ability remains limited. When the geological characteristics of the target region differ substantially from those in the training domain, prediction accuracy declines, indicating that the model is sensitive to spatial distribution biases in the training data. Furthermore, the fixed local receptive field of convolution layers constrains the network’s ability to capture long-range spatial dependencies and global seafloor structures, which may hinder accurate representation of complex bathymetric patterns. Future studies may consider employing architectures with enhanced global context modules or multi-scale hybrid designs, and incorporating higher-resolution more diverse data to improve model interpretability, robustness, and cross regional transferability.

## Resource availability

### Lead contact

Requests for further information and resources should be directed to and will be fulfilled by the lead contact, PhD, Jinyun Guo (jinyunguo1@126.com).

### Materials availability

This study did not generate new unique reagents.

### Data and code availability

• Predicted bathymetry data have been deposited at Zenodo and are publicly available as of the date of publication. The repository information is listed in the key resources table.
• This paper analyzes existing, publicly available datasets from multiple repositories. All datasets are publicly accessible as of the date of publication, and the corresponding repository information is listed in the key resources table.
• All original code has been deposited at Github and is publicly available as of the date of publication. The repository information is listed in the key resources table.
• Any additional information required to reanalyze the data reported in this paper is available from the lead contact upon request

## Acknowledgments

The authors are grateful to SIO for providing the marine deflection of the vertical model, vertical gravity gradient model and bathymetry model data, Shandong University of Science and Technology for sharing the SDUST2022GRA model, the Technical University of Denmark for providing MDT and MSS model, the International Hydrographic Organization for providing GEBCO_2024 model, the National Centers for Environmental Information for providing sedimentary thickness and single-beam ship-borne bathymetry data. This research is supported by the 10.13039/501100001809National Natural Science Foundation of China under grants 42430101, 42274006, and 42192535.

## Author contributions

Conceptualization, Jia Guo and Jinyun Guo; methodology-implementation, Jia Guo; investigation, Jia Guo; writing-original draft, Jia Guo; All authors commented, edited, and revised the final manuscript; funding acquisition, Jinyun Guo; supervision, Jinyun Guo.

## Declaration of interests

The authors declare no competing interests.

## STAR★Methods

### Key resources table

**Table:** 

REAGENT or RESOURCE	SOURCE	IDENTIFIER
**Deposited data**		

Predicted bathymetry data	This paper	https://doi.org/10.5281/zenodo.15873338
Code for models	This paper	https://github.com/JiaGUO1997/Bathymetry#
Single-beam ship-borne bathymetry	National Centers for Environmental Information	https://www.ncei.noaa.gov/maps/bathymetry/
SDUST2022GRA	Shandong University of Science and Technology	https://doi.org/10.5281/zenodo.8337387
VGG_V32.1	Scripps Institution of Oceanography	https://topex.ucsd.edu/pub/global_grav_1min/
DOV_V32.1	Scripps Institution of Oceanography	https://topex.ucsd.edu/pub/global_grav_1min/
DTU21MSS	Technical University of Denmark	https://ftp.space.dtu.dk/pub/DTU21/1_MIN/
DTU22MDT	Technical University of Denmark	https://ftp.space.dtu.dk/pub/DTU22/MDT/
GlobSed-v3	National Centers for Environmental Information	https://www.ncei.noaa.gov/products/total-sediment-thickness-oceans-seas
Topo_25.1	Scripps Institution of Oceanography	https://topex.ucsd.edu/pub/global_topo_1min/
ETOPO_2022	National Centers for Environmental Information	https://www.ngdc.noaa.gov/thredds/catalog/global/ETOPO2022/30s/30s_bed_elev_netcdf/catalog.html?dataset=globalDatasetScan/ETOPO2022/30s/30s_bed_elev_netcdf/ETOPO_2022_v1_30s_N90W180_bed.nc
GEBCO_2024	Nippon Foundation and General Bathymetric Chart of the Oceans	https://www.gebco.net/data-products/gridded-bathymetry-data

**Software and algorithms**		

Python (v3.8.11)	Python Software Foundation	https://www.python.org
Pytorch (v2.0.0)	Linux Foundation	http://pytorch.org
GMT (v6.5.0)	School of Ocean and Earth Science and Technology	https://www.generic-mapping-tools.org/

### Method details

#### Ship-borne single-Bream tracks

This paper used single-beam ship-borne bathymetry data obtained from the National Centers for Environmental Information (NCEI) as the primary dataset. The dataset includes 583 ship-borne single-beam tracks, with a total of 5 183 703 measurement points.

However, because the bathymetric data were collected over an extended period some of the measurements obtained before the advent of the global positioning system often suffered from poor georeferencing and significant measurement errors.^41^ Therefore, it is necessary to pretreat the bathymetric data to eliminate measurement points that share identical coordinates or contain substantial errors. A high-accuracy bathymetric model was used as a reference to ensure the reliability of the ship-borne bathymetric measurement. The Topo_25.1 model,^38^ a 1′×1′ resolution bathymetric model, has an STD of about 435 m, reflecting its overall accuracy for large scale bathymetry mapping.^34^ This model serves as a prior reference to identify and filter out points with significant errors. The preprocessing procedure mainly involves two parts:

The first part is to remove duplicate measurement points that share the same longitude and latitude coordinates. For locations with multiple depth measurements, the depth value is set with the mean of all the measurements at that coordinate. Approximately 6.71% of the bathymetric points were aggregated, leaving 4 835 906 unique points. The second part is to exclude the unique bathymetric points with significant errors. First, Topo_25.1 model is used to calculate the predicted bathymetry at each unique point with bicubic spline interpolation. Bicubic spline interpolation ensures continuity of both the first and second derivatives across the interpolation domain. This property leads to smoother results and reduces noise compared with simpler interpolation methods. Moreover, unlike linear interpolation, bicubic spline interpolation incorporates information from a larger number of neighboring grid points, which improves accuracy in capturing spatial variations. The difference between the actual measured bathymetry and the Topo_25.1 predicted bathymetric values at each point is then computed, and the STD of these differences is obtained. To ensure robust outlier removal, unique bathymetric points with absolute bathymetric errors exceeding three times the STD were iteratively removed in three consecutive rounds. Finally, 5.23% of the unique points were removed, leaving 4 584 296 ship-borne single-beam bathymetric points.

The remaining points were partitioned into 5 ° × 5 ° latitude-longitude regions. Points within each region were independently and randomly allocated to training, validation, and test subsets in a 6:2:2 ratio, yielding a total of 2 750 561 training points, 916 841 validation points, and 916 894 test points.

#### Marine geodetic data

The gravity anomaly represents variations in the Earth’s gravity field due to subsurface density contrasts and seabed topography.^18^ The gravity anomaly used in this study comes from the SDUST2022GRA model^42^ developed by Shandong University of Science and Technology with a spatial resolution of 1 arcmin, which was developed and released in 2022^43^ (Figure 4A). This model is developed by determining cross-track geoid gradients and integrating both along-track and cross-track geoid gradient information of radar and laser altimeter data.

VGG can reflect the influence of the under objects or seabed topography on the local gravity field, while DOV reflects the tilt of the gravity vector caused by local mass anomalies (such as seabed ridges or basins). Both VGG and DOV have been widely used to predict the bathymetry in multiple studies.^34^^,^^35^^,^^37^^,^^44^ In our study, these data VGG_V32.1 (Figure 4B) and the meridian and prime components of DOV_V32.1 (Figures 4C and 4D) are derived from version 32.1 released by the Scripps Institution of Oceanography (SIO) in 2022, with a spatial resolution of 1 arcmin.

MSS represents the temporal average of the absolute sea surface height. The MSS model used in this study is the DTU21MSS model,^45^^,^^46^ developed by the Technical University of Denmark, computed over the 1993–2012 period from multiple satellites. MDT describes the difference between the MSS and the geoid and reflects the mean circulation in the oceans. The MDT model used in this study is the DTU22MDT model, also developed by the Technical University of Denmark,^47^ derived using the DTU21MSS model.

Sedimentary thickness refers to the vertical accumulation of sediments over geological timescales on the seafloor or within sedimentary basins.^48^ It serves as a key indicator of sedimentary processes, tectonic evolution, and depositional environments. Recent studies^36^ have demonstrated that seafloor sediment effects are significantly connected to bathymetric inversion accuracy. GlobSed-v3 dataset^48^ provides high-resolution estimates of total sediment thickness by integrating seismic reflection profiles, drilling data, and geological mapping.

Collectively, these geodetic datasets provide complementary constraints on seafloor morphology at different spatial and physical scales. Gravity anomalies and their derived products, such as VGG and DOV, capture both large-scale tectonic features and short-wavelength structures associated with seamounts, ridges and fracture zones.^49^ MSS and MDT incorporate altimetric information that reflects the underlying seafloor topography and ocean circulation. Sediment thickness provides long-term geological constraints that influence bathymetric inversion accuracy.^50^ Integrating these diverse datasets as CNN inputs allows the model to learn multi-scale, multi-physics representations of seafloor characteristics, thereby improving the reliability of bathymetric prediction.^35^^,^^36^^,^^37^

#### Existing bathymetric models

To support the preprocessing of the measurement points and evaluate the accuracy of the developed method, this study introduces the Topo_25.1 model at a 1 arcmin resolution. Additionally, the higher resolution GEBCO_2024 model is employed exclusively for validation purposes.

The Topo_25.1 model^38^ is a global bathymetric model released in 2023 by SIO as version 25.1. It provides coverage from 80° N to 80° S and has a spatial resolution of 1′ × 1′.

The ETOPO_2022 model,^39^ released in 2022 by the NCEI of National Oceanic and Atmospheric Administration (NOAA), is a seamless global digital elevation model that integrates topography and bathymetry. It provides continuous global coverage at a spatial resolution of 15 arc-seconds, and also the version of 30 arc-seconds and 60 arc-seconds.

The GEBCO_2024 model,^40^ released in July 2024 by the Nippon Foundation–GEBCO Seabed 2030 Project, is the latest and most up-to-date global elevation dataset currently available. Jointly developed by the Nippon Foundation and General Bathymetric Chart of the Oceans (GEBCO), it provides continuous coverage from 90° N to 90° S at a spatial resolution of 15 arc-seconds, integrating both land elevation and ocean bathymetry. The model is based on SRTM15 + v2.6^51^^,^^52^ between latitudes of 50°S and 60°N and incorporates regional multi-beam data compiled by the four Seabed 2030 Regional Centers of National Institute of Water and Atmospheric Research from New Zealand, using a remove-restore blending technique to ensure seamless integration. Owing to this comprehensive integration of high-quality regional measurements, the GEBCO_2024 model demonstrates superior performance compared to other existing models.

Since the CNN in this study is trained to predict residuals relative to Topo_25.1, comparison with Topo_25.1 is indispensable, as it directly evaluates the improvement of our model. In addition, we have included two widely used global bathymetry models, GEBCO_2024 and ETOPO_2022, as supplementary benchmarks. This setup allows us to assess the robustness and competitiveness of our predictions relative to current international standards.

#### Data organization

The organization of the input and output of a CNN affect not only training and prediction accuracy but also determines the choice of neural network architecture. In this study, the input data are structured as an 8 × 8 grid at 1′×1′ spatial resolution, with 13 channels, each representing different geographical or physical characteristics (Figure 5).

The first two channels represent the differences in longitude (ΔB) and latitude (ΔL) between the training target and the grid points, providing spatial location information. The third channel contains normalized bathymetric depth data (H) from Topo_25.1 model by dividing the original values by 1000. The fourth channel distinguishes land from sea based on bathymetric depth, assigning a value of 1 to sea areas and 0 to land, to help solve the prediction of the shallow water near the land. The gradient of bathymetric depth and its direction computed using the Scharr operator and presented via the arctangent function, are also included to show the changing trend of the bathymetry. Scharr operator is an edge detection operator, usually for the gradient calculation. It includes matrices in two directions, horizontal and vertical, and performs matrix dot multiplication operations on image data in the corresponding directions. The mean of the dot product results is used to represent the gradient of the center point of the matrix, which is used as the gradient data of the pixel at the corresponding position of the image. The common form of the Scharr operator under the 3 × 3 kernel is as follows:

$$G_{x}=\left[\begin{array}{ccc} -3 & 0 & +3\\ -10 & 0 & +10\\ -3 & 0 & +3 \end{array}\right],G_{y}=\left[\begin{array}{ccc} -3 & -10 & -3\\ 0 & 0 & 0\\ +3 & +10 & +3 \end{array}\right]$$

Where *G*_*x*_ could detect the horizontal intensity variation, and *G*_*y*_ for the vertical variation. And the magnitude and direction could be presented as:

$$magnitude=\sqrt{{G_{x}}^{2}+{G_{y}}^{2}}$$

$$direction=\arctan\left(\frac{G_{y}}{G_{x}}\right)$$

Additional input channels include the gravity anomaly (Δg), VGG, MDT, the meridian (ξ) and the prime (η) components of the DOV, the MSS and log-transformed sedimentary thickness. The integration of these diverse geophysical variables improves the model’s ability to capture rich and complex characteristics of oceanic environment.

Due to the inconsistent grid nodes among multiple datasets, to minimize potential distortion caused by excessive interpolation, we adopt a “minority follows the majority” principle. Although all datasets are constructed in grids, they are not aligned on the same grid system. For example, the prime/meridian DOV, VGG, MSS and Topo_25.1 model used grids starting from positions offset by 15″, whereas other datasets start exactly at integer grid points. To ensure consistency, we adopted the grid system used by the majority of datasets, and the minority datasets were interpolated and adjusted accordingly. This principle of “minority follows the majority” ensures that all datasets are mapped to a unified grid for subsequent training and analysis. Specifically, we use continuous surface splines to resample all models onto a unified grid starting from 0°30″N, 120°30″E with interval 1′.

#### Network design

A CNN was implemented to predict bathymetry. The model’s architecture (Figure 5) consisted of three two-dimensional (2D) convolutional layers, each followed by a Leaky ReLU activation function and a 2D max pooling layer. Following the convolutional layers, the feature maps were flattened and passed through four fully connected layers consisting of 64, 128, 128 and 1 neurons, respectively. Batch normalization and Leaky ReLU activation were applied after each fully connected layer, except for the final output layer. The input to the model consists of multi-channel data with dimensions of 8 × 8 × 13. The CNN was trained with a batch size of 2048 over 100 epochs using the Adam optimizer with an initial learning rate of 1 × 10-3 and a learning rate decay factor of 0.1 applied after 10 epochs of stagnation. Both the training and validation sets were used to optimize the model’s hyperparameters. Once the model was successfully trained, it was used to predict bathymetric depth at all grid nodes spaced at 1′ intervals, starting from (0°N, 120°E) in latitude and longitude, thereby generating a structured grid of bathymetry estimations. The corresponding prediction of each test points was generated using bicubic spline interpolation method based on the grid. An example of these grid points is indicated by the red circles in Figure 5.

The network will predict the residual between the seafloor depth of training point and a reference point which is the upper-left corner of arcmin cell from Topo_25.1 model. To train the network, we employed an MSE loss function. The loss is defined as:

$$L=\frac{1}{N}\sum_{i=1}^{N}{\left(y_{i}-\hat{y_{i}}\right)}^{2}$$

Here, *y*_*i*_ and $\hat{y_{i}}$ represent the ground truth and predicted values, respectively, *N* is the number of samples.

The implementation was carried out using Python (v3.8.11; Python Software Foundation, Wilmington, DE, USA) and pytorch (v2.0.0). Training was performed on an NVIDIA GeForce RTX 3080 GPU (NVIDIA, Santa Clara, CA, USA). The total training time was approximately 120 h.

### Quantification and statistical analysis

All statistical evaluations were conducted using Python (v3.8.11). The network’s performance was statistically evaluated by studying the differences between the predicted and the reference depth values. To assess the accuracy of the developed method, the following metrics were also employed: RMS, MAE and R^2^.

RMS and MAE represent the average magnitude of prediction errors, irrespective of their direction. Lower RMS and MAE values indicate higher predictive accuracy and consistency, both of which are essential for reliable reconstruction of bathymetry. R^2^ quantifies the proportion of variance in the reference depth value explained by the predicted outputs. The analysis details are provided in the Tables 1, 2, and Figure 3.

In addition to pointwise error metrics, spatial consistency and regional stability were qualitatively evaluated through visual inspection of predicted depth maps. However, these qualitative assessments fall outside the scope of the quantitative analysis presented here.
